# Health data sharing in Germany: individual preconditions, trust and motives

**DOI:** 10.3389/fpubh.2025.1538106

**Published:** 2025-02-05

**Authors:** Elias Kühnel, Felix Wilke

**Affiliations:** Chair of Sociology in Social Work, Department of Social Work, University of Applied Sciences, Jena, Germany

**Keywords:** health data, data sharing, trust, willingness, digitalization, privacy

## Abstract

**Objective:**

The use of health data plays an important role in medical research. In Germany, the secondary use of health data is not yet as established as in other countries. In order to exploit the full potential of using health data, a key prerequisite is that a large part of the population is willing to share their health data. The aim of this study is to identify attitudes and factors that influence citizens’ willingness to share personal health data for medical research purposes.

**Methods:**

A standardized cross-sectional telephone survey was conducted among the German population aged 18 and above. The representative survey of a total of 1,004 people took place from December 7th to 21th, 2023. Multiple linear regression analyses were conducted to identify potential factors influencing the willingness to share health data.

**Results:**

Around 43% of respondents are willing to share their health data with public research institutions, while around 17% are still undecided. The population, however, is particularly skeptical about data being shared with commercial companies. Less than a quarter of respondents (23%) would share their data with small and medium-sized enterprises and only 15 percent with technology companies. Regarding the factors influencing the willingness to share data with research institutions, three overarching factors can be named: (1) individual characteristics, in particular the level of education and technical competence, (2) individual motives, such as altruism as well as worries and concerns regarding health data sharing, and (3) trust in institutions, especially trust in science and technology companies.

**Discussion and conclusion:**

Although the study shows a general openness towards sharing health data and underlines the importance of altruistic motives, social inequalities and concerns about health data sharing are also evident. Trust plays a particularly important role here, as it acts as a mediator between individual preconditions and individual motives. In order to exploit the full potential of secondary data use in medical research, comprehensive information of the public about data sharing and use as well as transparent and open communication are required to strengthen trust in the actors involved and the data sharing process.

## 1 Introduction

### 1.1 Background

In recent years, the role of health data sharing has become increasingly important for advancing research in public health and medical sciences. Access to personal health data enables researchers to analyze trends, develop new treatments, medications or medical devices and inform policies that can improve healthcare delivery on a broad scale. In contrast to other countries like the Scandinavian Countries or Australia, the secondary use of health data for research purposes is yet not possible on a large scale in Germany. Until the year 2024 Germany lacks a technical infrastructure to collect and handle large scale of health data. Furthermore, in Germany privacy concerns are widely spread. However, the Federal Ministry of Health has introduced a new legislative framework that aims at health data sharing for a large part of the population via the new Electronic Health Record (EHR) (1, 2). Understanding the barriers and facilitators to health data sharing in Germany is essential to harnessing the full potential of health data for societal benefit. Especially important are potential socioeconomic and educational disparities, and differing levels of digital literacy. They may lead to a divide in digital health (3) and confine potential benefits of health data sharing, as important groups could be excluded. This article unravels the overall acceptance of health data sharing for medical research purposes in Germany and identifies factors determining differences in health data sharing. It focuses on the special role of trust.

### 1.2 State of research

*The sharing of health data* for research purposes can take place in many different contexts. These range from publicly managed Electronic Health Records (4) to data sharing through smart devices such as wearables (5). Research on data sharing has shown that a significant proportion of the population (often more than 50%) is willing to share their health data (5–10). Notably, these results are stable across studies that focus on public and private contexts of data sharing. However, there are differences between countries. For example, Scandinavian countries show the highest willingness to share health data, while people in Southern Europe are more skeptical about health data sharing (11). Some studies show very high rates of agreement. In Germany, a recent survey found that 8 out of 10 citizens are willing to share their health data for scientific purposes (12, 13). However, such high acceptance rates are likely to be biased by positive wording in the questionnaire and the use of online surveys.^1^

International research has already pointed out several factors determining the willingness to share health data. A critical aspect of the decision to share personal health data lies in individual *preconditions*, such as educational background, technical competence, and socioeconomic status. Additionally, individual *motives* – including altruistic considerations and personal benefits, such as advancements in healthcare – also impact data sharing attitudes. Yet, concerns over data privacy and misuse remain strong deterrents, particularly when data sharing involves commercial entities rather than public research institutions.

#### 1.2.1 Preconditions for data sharing

Studies in digital health have shown that the benefits and risks of a digitized health sector are not evenly distributed across society. On the one hand, socioeconomic inequalities are reflected in the health sector. On the other hand, technological tools and the ability to use them properly become more important with the digitalization of the health sector. Some scholars have warned that this development could lead to a new digital divide, with lower quality of health and higher risks for people with less experience with digital technologies (3, 14). Empirical evidence supports this hypothesis. The willingness to share personal health data is higher among people with higher socioeconomic status: Individuals with higher levels of education (5, 9, 15–18) and income (15) and those living in urban areas (9, 18) are more likely to share data. Regarding age, studies find ambiguous results. While some findings show a higher willingness to share health data among younger people (17, 18), others suggest a u-shaped relationship, with younger and older people showing a higher willingness, while middle-aged people are more skeptical about sharing health data (5, 16). Thus, there is clear evidence that the willingness to share health data depends on a person’s socioeconomic context. Digital literacy can be seen as another prerequisite. The “skills necessary to be able to live within a society in which communication is increasingly based on new technologies” include the ability to use new technologies and to process digital information (19). There are two important arguments why people who are more familiar with digital tools can be expected to share data more often: On the one hand, people who routinely use digital tools are better able to assess the risks and opportunities of sharing data. On the other hand, there is likely to be a habituation effect as technology companies like Google and Apple force people to share data in everyday life. Consistent with these arguments, empirical studies show that the more digitally savvy people are, the more likely they are to share health data (15, 18, 20–23).

#### 1.2.2 Motives

A second strand of research focuses on the motives that increase or decrease the willingness to share health data. The motives can be divided into altruistic and cost–benefit considerations. It can be expected that people who hope for health improvements (for society or for the individual) will be more willing to share their health data. These benefits must be weighed against the potential risks of sharing personal health data. Empirical studies show that motives play an important role in data sharing. The greater the potential benefits, including more altruistic ones such as advances in science and public health, the greater the willingness to share health data [e.g., (7, 8, 17, 20, 22, 24, 25)]. Not surprisingly, higher perceived risks reduce the willingness to share health data [e.g., (5, 15, 24)]. Potential benefits and risks have been empirically assessed in multiple ways (e.g., multimorbidity, health status, perceived risk of discrimination, technological risks, risk of being stigmatized) (26). Consistent with this, privacy (5, 7, 8, 20, 26), anonymity (7, 8, 20), and transparency (7, 8, 15) have also been shown to be successful means to encourage data sharing.

#### 1.2.3 Trust

This study argues, that a third important factor which is often neglected needs special attention: *trust*. In particular, there is limited understanding of how varying levels of trust in public versus private institutions influence data sharing decisions, especially given Germany’s stringent privacy regulations and strong data protection culture. From a sociological perspective trust plays a critical role in the health sector. Sociological analysis has shown its essential role in health in general (27). But it can also be considered a key mechanism for understanding the willingness to share health data. Most people are unable to properly assess the promises and risks of health data sharing. People looking for answers to questions such as “Will my health data help to improve health?” or “What are the chances that my health data will be de-anonymized or leaked?” have to rely heavily on expert knowledge. At the same time, this expert knowledge is far from secure. The risks of data sharing, as well as the potential benefits, depend heavily on other actors and can hardly be known in advance. For example, there is no guarantee of medical progress, and it is unclear whether the people sharing data will benefit from it. Similarly, the risks of data sharing can only be assessed in the context of existing knowledge and technology. Accordingly, new technologies in the field of Artificial Intelligence (AI) pose new challenges for data security (28) that have hardly been considered. When surveys stress the importance of privacy and anonymity for data sharing, these concepts must not be interpreted in terms of mere technical solutions. In a modern, complex world, trust is a necessary intermediary between people’s health data and the institutions using it. Although trust has not been the focus of research on health data sharing, those studies that have tested trust have demonstrated its important role. They can empirically show that trust in the institutions involved in data sharing is relevant, as is general trust in science (8, 15, 18, 20–22).

This study seeks to examine the influence of trust and the interplay of trust with individual factors and motives on the willingness to share health data for medical research within Germany’s population. Using a cross-sectional telephone survey, we analyze how factors such as individual preconditions, motives, and institutional trust affect data sharing attitudes.

## 2 Methods

### 2.1 Study design and data collection

The survey was designed as a cross-sectional study and was based on a representative randomized stratified random sample (29). A computer-assisted telephone interview (CATI) was used, taking into account both landline (30%) and mobile phone numbers (70%) (dual frame approach). Mobile phone connections enable a more precise and representative distribution of the population by age and gender than landline connections. For this reason, the decision was made in favor of a larger mobile phone sample. The mobile phone numbers were sampled stratified by telephone provider. It is not possible to regionally control the mobile phone sample in advance. The landline sample was stratified by federal state. In order to ensure the quality of the sample, it was compared with the population for age, gender and region during the field period. Statistics from the 2019 German microcensus served as the data basis to compare the sample distribution with the distribution of the characteristics in the population. People aged 18 and above with permanent residence in Germany were surveyed. The survey took place between December 7 and 21, 2023. The average interview duration was 21.5 min.

### 2.2 Survey instrument

The standardized questionnaire consisted of 32 questions and covered a total of eight topic areas: (1) digital literacy, tech affinity and tech anxiety, (2) willingness to share personal health data, (3) general attitudes and preferences towards health data sharing, (4) motivation to share health data, (5) attitudes towards digital technologies and trust in institutions as well as in data protection and privacy, (6) willingness to share health data and attitudes towards the electronic health record (EHR), (7) donation to charitable causes, and (8) general information about the person and health status. Most questions were based on a 5-point Likert scale or binary response options and included multiple response categories. The order of response options can significantly impact responses (30). To avoid possible distortions due to the order of the response categories, the majority of the questions were randomized. This approach was chosen to ensure that no systematic effects arise from the presentation of response options. The survey instrument was tested in two separate pretests. Firstly, a qualitative-cognitive pretest was carried out by telephone interviews. The so-called think-aloud technique was used, as well as specific questions to understand individual issues, terms and scales. A second pretest was carried out by the market and opinion research institute *drei.fakt* in Erfurt, which was commissioned to carry out the data collection. Randomly selected people were interviewed by telephone. In this pretest, the survey instrument was tested for its practical suitability in the field.

### 2.3 Data analysis

Uni- and bivariate descriptive statistics were used to characterize the distributions of the individual survey variables. To investigate the factors influencing the willingness to share health data for medical research purposes, a multiple linear regression analysis was conducted. For this purpose, various models were calculated in which additional covariates were successively integrated. The following question served as the dependent variable:

Would you be willing to share your health data anonymously for medical research purposes with the following institutions?

The question included a 5-point Likert scale from “yes, definitely” to “no, definitely not” and consisted a total of five items: (1) with public research institutions, (2) with private research institutions, (3) with technology companies (e.g., Google, Facebook, Apple), (4) with small and medium-sized companies, and (5) with health insurances. To reflect the willingness to share health data for medical research purposes with research institutions in general, a mean index was created from the first two response categories (willingness to share health data with public and private research institutions), ranging from 0 (“no, definitely not”) to 4 (“yes, definitely”).

In preparation for the multivariate data analysis, measures of the internal consistency of scales (Cronbach’s Alpha) and exploratory factor analyses were conducted to identify determinant variables. When selecting the final covariates in the multivariate data analysis, theoretical considerations and previous empirical findings based on a literature review were taken into account. All models were also tested with an ordered logistic regression (ologit). The data were analyzed by Stata, version 17.0. In order to adequately represent the German population in terms of demographic, socioeconomic and geographical characteristics, the data were weighted by gender, age group, educational level, country of birth (Germany/not Germany), region (East/West Germany) and community size. For this purpose, a weighting variable was created using an iterative proportional fitting process (IPF). The reference data used were the German population statistics published by the Federal Statistical Office (DESTATIS) as of December 31, 2022.

## 3 Results

### 3.1 Sample characteristics

Out of the 1,004 respondents, 51.2% were female (*n* = 514), 48.8% were male (*n* = 489), and one respondent (0.1%) was of another gender (*n* = 1) (Table 1). A majority of respondents (in total 57.3%) were 50 years old and older (50–59: 19% (*n* = 191); 60–69: 19.9% (*n* = 200); 70 and older: 18.3% (*n* = 184)), while 42.7% were younger than 50 years (18–29: 15.3% (*n* = 154); 30–39: 12.85% (*n* = 129); 40–49: 14.5% (*n* = 146)). The average age was about 51.7 years, ranging from 19 to 88 years of age. In addition, a majority of 58.8% had a highest educational qualification of up to a higher secondary school diploma (lower secondary school diploma: 15.4% (*n* = 209); higher secondary school diploma: 36.9% (*n* = 370)), while 19.2% had a high school diploma (*n* = 192) or a university diploma (23.1%, *n* = 231). 5.4% of the respondents were born in a country other than Germany (*n* = 54). A majority of respondents lived in medium-sized (26.1%, *n* = 257) or large cities (32.2%, *n* = 317), while 20.1% lived in rural areas (*n* = 198) and 21.7% in smaller cities (*n* = 214).

**Table 1:** Characteristics of the sample (*N* = 1,004).

	*n*	% (Unweighted)	% (Weighted)^a^
Sex			
male	489	48.71	49.21
female	514	51.20	50.72
diverse	1	0.10	0.07
Age group			
18–29	154	15.34	15.38
30–39	129	12.85	15.59
40–49	146	14,54	14.52
50–59	191	19.02	17.48
60–69	200	19.92	16.52
70 and older	184	18.33	20.52
Educational level			
Lower secondary school diploma	209	20.86	29.67
Higher secondary school diploma	370	36.93	31.70
High school diploma	192	19.16	17.58
University diploma	231	23.05	21.05
Country of birth			
Germany	940	94.57	86.05
Other	54	5.43	13.95
Region			
East Germany	141	14.04	14.93
West Germany	863	85.96	85.07
Community size			
up to 5,000 inhabitants	198	20.08	13.63
5,000 to less than 20,000 inhabitants	214	21.70	26.55
20,000 to less than 100,000 inhabitants	257	26.06	27.60
100,000 inhabitants or more	317	32.15	32.23
Total	1,004	100.00	100.00

a Data weighted by age group, gender, region (East/West Germany), country of birth, educational level and municipality size.

After weighting the data, underrepresented groups were given a higher weighting. This applies to male respondents, people under 40 years of age and those aged 70 and above. In addition, the weights of people with a lower secondary school diploma, people who were not born in Germany, people living in East Germany and urban areas with 5,000 or more inhabitants were revised upwards.

### 3.2 Willingness to share health data for research purposes

**Figure 1:**
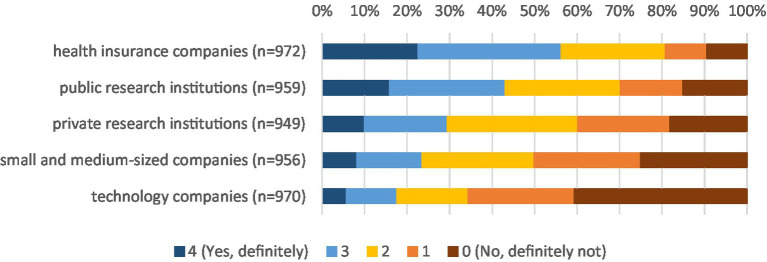
Willingness to share health data by data user. * Data weighted by age group, gender, region (East/West Germany), country of birth, educational level and municipality size.

The survey shows that the population is in principle open to data sharing. The highest willingness to share personal health data for medical research purposes can be observed with health insurances at around 57% (Figure 1). About 43% of respondents are willing to share their health data with public research institutions (30% would not share, 17% are still undecided), while only about 29% would share their data with private research institutions. In the case of private research institutions, it is also apparent that a relative majority of around 40% would refuse to share their data (29% would share, 31% are undecided). In contrast, a large majority of respondents is unwilling to share their health data with commercial companies (small and medium-sized companies: 50%), especially with technology companies (66%).

### 3.3 Factors influencing the willingness to share health data

**Figure 2:**
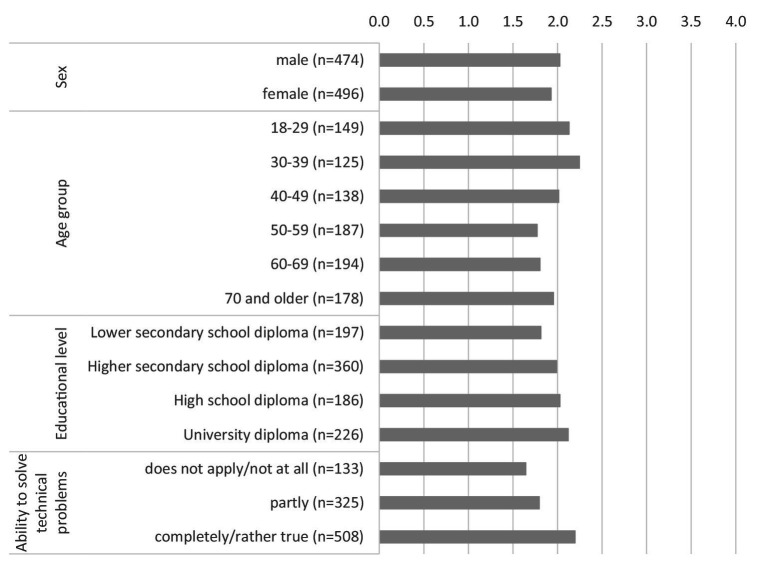
Willingness to share health data with public and private research institutions by individual characteristics (0 = no, definitely not, 4 = yes, definitely). * Data weighted by age group, gender, region (East/West Germany), country of birth, educational level and municipality size.

Figure 2 shows the willingness to share health data with public and private research institutions depending on demographic and socioeconomic characteristics, but also on technical competence. The findings are rather moderate in terms of demographic and socioeconomic characteristics. Regarding gender, men show a slightly higher willingness to share data. The distribution across age groups is inhomogeneous. While younger and middle-aged people up to 49 years of age and those aged 70 and older in particular show a relatively high willingness to share health data, people between the ages of 50 and 69 are the least willing to share their health data. These findings are similar to those of Hirst et al. (5) and Weng et al. (16). The higher willingness to share health data among people aged 70 and over could be related to the fact that these people may have a greater interest in medical progress and are more likely to benefit from it themselves due to an increase in morbidity in older age and more extensive contact with health care actors, such as physicians. In contrast, there is a linear relationship when it comes to educational level. It can be observed that the higher people’s educational level, the more willing they are to share their health data. It can also be seen that people who have a better assessment of their ability to solve technical problems independently are more willing to share their health data.

**Figure 3:**
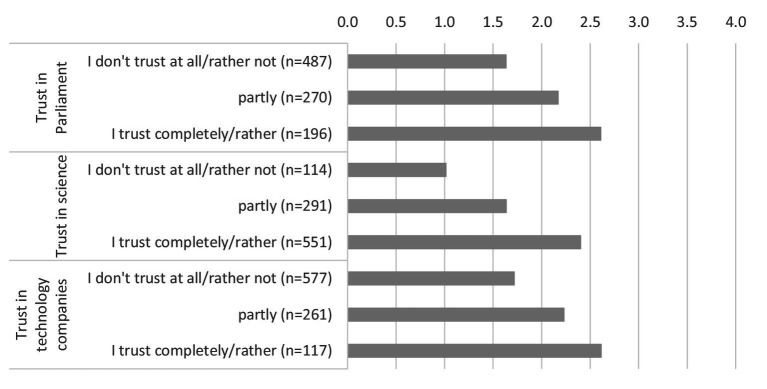
Willingness to share health data with public and private research institutions by institutional trust (0 = no, definitely not, 4 = yes, definitely). * Data weighted by age group, gender, region (East/West Germany), country of birth, educational level and municipality size.

The willingness to share health data depending on trust in institutions is shown in Figure 3. We distinguish between trust in parliament, in science and in technology companies. It becomes apparent that the willingness to share personal health data with public and private research institutions increases with increasing trust in institutions. Linear relationships can be observed in all three institutions. The differences in trust in science are particularly striking. This is where the biggest differences can be seen between people with low trust in science and those with high trust.

**Figure 4:**
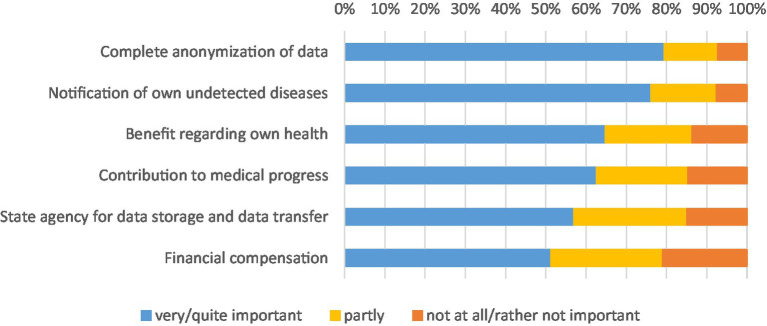
Importance of individual motives. * Data weighted by age group, gender, region (East/West Germany), country of birth, educational level and municipality size.

In our study, we also asked participants how important certain aspects are to them when sharing their health data for medical research purposes (Figure 4). The results show that the respondents place a particularly high value on maintaining their anonymity by sharing their health data. Around 79% state that complete anonymization of their data is very or somewhat important to them. However, intrinsic motives also play an important role, such as individual benefits. For example, 76% of respondents stated that they would like to be notified of own undetected diseases. This highlights a tension between participants’ desire for personalized feedback on unrecognized health problems and the principles of anonymization that prevent individualized communication afterwards. Around 65% said that they hoped that sharing their health data would benefit their own health. Financial compensation, on the other hand, is less important to the respondents, but here too, a small majority of around 51% say that financial compensation is very or somewhat important to them. In addition to individual benefits, altruistic motives also play a major role. Around 63% of respondents consider it very or somewhat important to make a contribution to medical progress by sharing their health data for medical research purposes. There is also a strong desire for a state agency to store and transfer data. A majority of 57% rate this as very or somewhat important.

To investigate the influence of potential factors on the willingness to share data, multiple linear regressions were calculated. These include various models into which additional covariates were successively included (Table 2). The dependent variable is the willingness to share health data with public and private research institutions (calculated as the mean of both statements). The first model refers to demographic and socioeconomic characteristics as well as the health status (chronic or mental disease). The second model included digital and technical competence. This involves the frequency of Internet use and the perceived ability to solve technical problems independently. Trust in institutions was added in the third model. Here, trust in parliament, in science and in technology companies was taken into account. In the last model, additional variables were added that relate to intrinsic motives, specifically the individual importance of making a contribution to medical progress or of receiving personal benefits for the own health by sharing personal health data for medical research purposes. Worries or concerns about data sharing were also addressed. Firstly, a variable was included that relates to the concern that respondents could be disadvantaged by their health insurance as a result of sharing their health data. Worries about unauthorized sharing of the data with third parties were also taken into account. The final model also included a variable that relates to the general willingness to donate to charitable causes. For all models, a significance level of less than 5% was assumed.

**Table 2:** Multiple linear regression: willingness to share health data with public and private research institutions (mean).

	Model 1	Model 2	Model 3	Model 4
	*β* (P > |t|)	*β* (P > |t|)	*β* (P > |t|)	*β* (P > |t|)
Sex (male)	0.0615 (0.476)	−0.0431 (0.621)	−0.0770 (0.302)	0.0365 (0.578)
Age	**−0.0074 (0.009)**	−0.0040 (0.164)	0.0007 (0.750)	−0.0005 (0.798)
Educational level				
Lower secondary school diploma	**−0.3229 (0.013)**	**−0.3029 (0.019)**	**−0.2643 (0.018)**	**−0.2372 (0.016)**
Higher secondary school diploma	0.0050 (0.967)	0.0284 (0.812)	−0.0140 (0.892)	−0.0340 (0.709)
High school diploma	−0.1128 (0.419)	−0.0550 (0.690)	−0.0838 (0.478)	−0.0248 (0.810)
University diploma	Reference	Reference	Reference	Reference
Household income	0.0000 (0.271)	0.0000 (0.279)	0.0000 (0.074)	0.0000 (0.996)
Country of birth (Germany)	0.0443 (0.743)	0.0652 (0.623)	0.1494 (0.188)	0.1546 (0.120)
Chronic disease	**0.2456 (0.011)**	**0.2491 (0.009)**	**0.1685 (0.039)**	0.0916 (0.198)
Mental disorder	**0.2407 (0.043)**	**0.2429 (0.038)**	**0.2680 (0.007)**	0.1284 (0.144)
Internet use (daily)		0.1296 (0.336)	0.1418 (0.221)	0.0536 (0.597)
Ability to solve technical problems		**0.2173 (0.000)**	**0.1359 (0.000)**	**0.1032 (0.001)**
Trust in the parliament			0.0559 (0.126)	0.0268 (0.403)
Trust in science			**0.4409 (0.000)**	**0.1924 (0.000)**
Trust in technology companies			**0.2192 (0.000)**	**0.1454 (0.000)**
Contribution to medical progress				**0.3962 (0.000)**
Benefit regarding own health				0.0739 (0.099)
Worries about disadvantages with health insurance				−0.1003 (0.149)
Worries that data will be disclosed to unauthorized third parties				**−0.1667 (0.031)**
Donation to charitable causes				0.0929 (0.166)
Constant	**2.2912 (0.000)**	**1.9022 (0.000)**	**1.5918 (0.000)**	**1.7117 (0.000)**
Adjusted *R*^2^	0.0319	0.0671	0.3195	0.4844
Pr > F	0.0002	0.0000	0.0000	0.0000
N	734	734	734	734

Data weighted by age group, gender, region (East/West Germany), country of birth, educational level and municipality size. Bold = statistically significant (*p* < 0.05). All models were also tested with an ordered logistic regression (ologit). The results remained unchanged. Only for the country of birth significant positive effects could be observed in models three and four and for benefit regarding own health a significant positive effect in model four.

The results show that demographic and socioeconomic characteristics have a rather moderate effect on the dependent variable. Only the level of education shows a consistent, statistically significant effect. The higher the level of education, the more willing people are to share their health data. This confirms the results for educational level with the previously mentioned bivariate relationships. In addition, there is a significant influence of technical competence. People with higher technical competence are therefore more willing to share their health data than people with lower technical skills.

The multivariate regression analysis also shows a large influence of trust in institutions. While we could not find any significant effects on trust in parliament, trust in science and technology companies shows that the willingness to share data increases significantly with increasing trust. This finding is particularly evident in trust in science, where the strongest effect can be seen. Since trust is measured on a 5-point scale like the dependent variable, the coefficient of trust in science (0.4409) indicates, that a person with low trust in science has a more than two points lower willingness to share health data (0.44*5), than a similar person with high trust.

Furthermore, the results show that people who want to contribute to medical progress by sharing their data are particularly willing to share health data. This underlines the special role of altruistic motives and prosocial framing in data sharing. However, the connection to medical research seems to be crucial, since no effect can be observed on the general willingness to donate to charitable purposes. It is also interesting to note that due to the inclusion of motivational factors the effects of chronic or mental diseases become insignificant. This suggests that people with pre-existing conditions may have a stronger motivation to contribute to medical progress because of their own personal involvement, but also that they may benefit more from the results of medical research. Finally, concerns about data sharing also have a significant impact on the willingness to share data. It can be observed that people who are worried about their health data being shared with third parties without authorization are less willing to share their data. Concerns about data protection and data security therefore appear to be an important motive. These concerns should therefore be addressed as they can significantly influence the decision to share health data.

## 4 Discussion

Our research findings, conducted by a telephone survey, support some important research findings regarding the willingness of health data sharing: On the one hand, respondents are open to sharing their health data for medical research purposes in general (5–10). However, as we discovered in our study, this only applies to public health insurances and public research institutions. In contrast, there is a high level of skepticism about sharing data with commercial companies, especially technology companies. This suggests that people have higher trust in public actors from healthcare and science than in commercial companies. The relatively high willingness to share health data with health insurances and public research institutions may reflect the perception that these organizations are more responsible and reliable in handling sensitive data. Commercial companies, especially technology companies, may be viewed with greater caution due to privacy concerns and profit motives. This finding partly explains the lower level of trust in commercial companies or private research institutions mentioned previously (31). On the other hand, our study also shows similarities with other findings regarding relevant factors influencing the willingness to share health data, in particular regarding institutional trust (5, 8, 15, 17, 18, 20–22, 24), educational level (9, 15–18, 23), technical competence (15, 18, 20–22), concerns regarding data protection and privacy (5, 15, 24), and beneficial and altruistic motives (7, 8, 17, 20, 22, 24, 25).

By applying a different survey methodology in contrast to the most widely used online surveys we can also show some relevant differences. Overall, our study shows a comparatively lower willingness to share health data than other national and international empirical findings suggest. There may be several reasons for this. First of all, the survey method plays a central role. As mentioned previously, online surveys have the disadvantage that the samples are often very selective, excluding important population groups, such as people without internet access or with low digital skills, especially older people or those with lower socioeconomic status (32). Current studies consistently demonstrate that internet usage and digital skills are strongly influenced by age and socioeconomic factors, with individuals with higher age [e.g., (33, 34)] and from lower socioeconomic backgrounds [e.g., (35, 36)] often having less access to digital tools and lower levels of digital competence. However, our study showed that people with low digital and technical skills and those with low levels of education are particularly reluctant to share their health data. This indicates the presence of a digital divide and educational inequalities, which are a major problem in the exchange of health data. Especially with regard to data quality, selective data sets pose a threat to the quality of medical research and its results. When conducting telephone surveys, it must be pointed out that certain interviewer effects cannot be completely ruled out. These could lead to respondents displaying socially desirable response behavior. In this case, the results would be expected to show a higher willingness to disclose health data. However, our results show that this is clearly not the case in this study compared to online surveys, which suggest an even higher willingness. Furthermore, we tried to use a strictly neutral question to measure the willingness to share health data, because the respondents’ answers depend largely on the wording of the questions, for example if they are framed positively. In contrast some other studies include positive outcomes of data sharing and thereby may exaggerate the willingness to share health data.

Based on our findings, three overarching factors influencing the willingness to share health data can be extracted:

Firstly, individual preconditions play an important role. It has been shown that socioeconomic characteristics in particular, especially the level of education, and technical competence have an important impact on the willingness to share health data. This could be an indication that knowledge and the ability to assess potential risks, but also opportunities, in relation to the use of health data for medical research play a crucial role. On the other hand, technology literacy could play an essential role in the willingness to share data, especially among younger people. This points to social inequalities and the presence of a digital divide that need to be urgently addressed, especially as they can lead to a high selectivity of the data and consequently to reduced data quality for medical research. This aspect is particularly relevant because older people and those with low socioeconomic status have an increased risk of morbidity (37, 38). This selectivity could become particularly problematic for research on new therapeutic options or new medications.

Secondly, individual motives are an important factor. On the one hand, these are altruistic or prosocial factors, as well as personal advantages in sharing health data, which seem to be particularly evident for people with pre-existing health conditions. This underlines the special role of altruistic motives and prosocial framing in health data sharing. On the other hand, worries and concerns about sharing health data also play a role, especially with regard to data security and data protection. This is also reflected in the respondents’ desire for anonymization and a state agency responsible for data storage and data sharing. These concerns should therefore be addressed as they can significantly influence the decision to share health data. It is therefore important to develop suitable strategies to minimize the worries and concerns of the population and to highlight the benefits of sharing health data. Transparent and open communication with the public is essential here. Creating secure anonymization procedures or clear regulations on data sharing are not enough. The population must be informed about what happens to their health data so that they can make an informed decision.

Thirdly, trust in institutions plays a key role. Sharing health data means acting in a highly socially uncertain situation, with little knowledge about technological and personal short- or long-term risks and benefits. It is therefore particularly important that the population has confidence that their health data will be handled safely and responsibly. These findings suggest that trust plays a more important role the more specific it is regarding the sharing of health data for medical research. Trust is crucial in two ways: On the one hand, trust comes into play where people are faced with uncertainty, for example due to existing knowledge gaps or difficulties in assessing risks and chances regarding the sharing of their own health data. On the other hand, trust plays a central role especially when people experience an antagonism between altruistic and beneficial motives and concerns regarding the sharing of health data. For example, if people want to contribute to medical progress or hope to gain personal benefits from sharing their health data, but are also concerned that the data may not be secure or they perceive risks that their data could be misused or passed on to unauthorized third parties, trust serves as a crucial mediator and ultimately determines the decision for or against sharing data.

Finally, some potential limitations of the study and comparable results of other studies need to be mentioned. Especially in Germany, the sharing of health data for secondary use is not as advanced as in other countries, such as Scandinavia, and the population may not yet have a comprehensive understanding regarding the topic. It can therefore be assumed that many of the respondents in this study (and comparable studies) have not yet given much thought to the issue of health data sharing. The answers should therefore be interpreted as first impulses for their current attitudes. It can be assumed that attitudes will change over time the more intensively the population is confronted with the issue, the more prior knowledge they have and the better they can assess the potential risks and opportunities associated with data sharing. Therefore, further research is necessary to address these aspects, especially once the framework conditions for secondary data use in Germany are more mature and the population has had initial experience with health data sharing. Future studies should consider longitudinal research to explore how public attitudes towards data sharing evolve with increased exposure to digital health systems and greater regulatory clarity around data protection.

A general methodological limitation in surveys is the limited ability to analyze non-response patterns. Telephone surveys, as well as other surveys, face an increasing amount of non-participation. A significant proportion of non-participation consisted of individuals who either showed no interest in participating or could not be reached. Reasons for refusal included general disinterest in the topic, lack of availability at the scheduled time, or reluctance to disclose personal data. This introduces the risk of an unknown bias, which is a common issue in studies of this kind.

## 5 Conclusion

This study underscores a nuanced view of health data sharing among the German public, revealing both a general openness to sharing data with trusted research institutions and marked skepticism towards sharing with commercial companies. Public trust in institutions, particularly sciences, is crucial for fostering willingness to share health data. The findings underscore the importance of creating transparent, secure frameworks for data management to create a context to build trust. The findings also highlight the need for targeted educational initiatives and outreach efforts to bridge the gaps in digital literacy and trust among diverse demographic groups. The findings show a digital divide that partly overlaps with educational disparities which both correlate with the willingness to share data. This suggests that information and public education should focus on different groups of the society in order to impede that predominantly individuals with higher socioeconomic status contribute to data-driven research. Otherwise less digitally literate groups may be underrepresented, potentially limiting the inclusivity and comprehensiveness of health research datasets. It is therefore necessary to ensure that information and educational initiatives are particularly accessible in order to adequately address people with lower digital skills and a low level of education.

For practitioners and policymakers, establishing clear, robust privacy measures and communicating these protections effectively to the public could alleviate concerns over data security, making individuals more comfortable with sharing their information. Additionally, fostering a culture of open dialogue and transparency regarding the uses and benefits of health data may increase public engagement, ultimately enriching the data pool available for medical research and supporting evidence-based advances in public health. The new legislation in Germany, that combines the switch to an opt-out for data-sharing via the Electronic Health Record and an information campaign by the statutory health insurances may not be sufficient to enable the population to make informed choices. In contrast, there may be many, especially among the less well educated, sharing data without even noticing. In addition to a simple, clear and target group-oriented approach, training courses and multimedia information initiatives could also be offered. Visual and auditory media, for example, containing videos, infographics or audio podcasts that provide information on the topic of data sharing would be conceivable. Especially at the beginning, it can also be advantageous that personal advice is available both analogue and digitally, so that the population can turn to trusted sources if they have any questions or encounter barriers. A scientific evaluation of the implementation of data sharing for research purposes in Germany, especially in the test phase, should be used to identify possible barriers and social inequalities and to promote acceptance and trust among different groups of the population.

## Data Availability

The datasets presented in this study can be found in online repositories. The names of the repository/repositories and accession number(s) can be found in the article/supplementary material.

## Footnotes

^1^Online surveys are more likely to produce biased results (39). In the context of health data sharing, the omission of people without Internet access is likely to bias the results. The cited survey also has a more positive framing of the question, emphasizing the positive effects of data sharing (12): “Assuming that in the future your personal health data, such as your medical history, examination results, X-ray images, etc. can be stored online in a digital health record. Would you agree that your personal health data is used anonymously and free of charge for the purpose of medical research so that in the future diseases can be better recognised and new treatments developed?”
